# Investigation of exacerbating factors for postpartum hair loss: a questionnaire-based cross-sectional study

**DOI:** 10.1097/JW9.0000000000000084

**Published:** 2023-06-16

**Authors:** Asuka Hirose, Masakazu Terauchi, Tamami Odai, Ayako Fudono, Kotoi Tsurane, Masaki Sekiguchi, Misako Iwata, Tatsuhiko Anzai, Kunihiko Takahashi, Naoyuki Miyasaka

**Affiliations:** a Department of Obstetrics and Gynecology, Tokyo Medical and Dental University, Yushima, Bunkyo, Tokyo, Japan; b Department of Women’s Health, Tokyo Medical and Dental University, Yushima, Bunkyo, Tokyo, Japan; c Department of Obstetrics and Gynecology, Tokyo Metropolitan Ohtsuka Hospital, Minami-Otsuka, Toshima, Tokyo, Japan; d Department of Biostatistics, M&D Data Science Center, Tokyo Medical and Dental University, Yushima, Bunkyo, Tokyo, Japan

**Keywords:** estrogen, inflammation, postpartum alopecia, questionnaire survey, suckling, telogen effluvium

## Abstract

**Background:**

Although postpartum hair loss is believed to be common, there is little reliable information.

**Objective:**

We sought to examine the factors that were associated with postpartum hair loss and to elucidate factors correlated with its pathogenesis.

**Methods:**

We carried out a questionnaire-based cross-sectional study. The study participants were women who delivered at 2 facilities and filled the questionnaire 10–18 months after delivery. The survey questionnaire included baseline characteristics, pregnancy details, delivery, childcare, and extent of postpartum hair loss. We divided participants into 2 groups according to the absence or presence of postpartum hair loss and performed logistic regression analyses.

**Results:**

A total of 331 (21.0%) responses were analyzed; among these 304 (91.8%) women had postpartum hair loss. The average time for the start, peak, and end of hair loss was 2.9, 5.1, and 8.1 months, respectively. Women with hair loss had an earlier time of delivery, a lower birth weight, a higher preterm labor rate, and longer-term breastfeeding. Logistical regression analyses revealed that longer-term breastfeeding and preterm labor were independent predictors of postpartum hair loss. The adjusted odds ratio for postpartum hair loss in women who ended breastfeeding 6–12 months postpartum versus those who ended it after 12 months or more was 5.96 (95% confidence interval [CI] [1.68, 21.09]) and 6.37 (95% CI [1.95, 20.76]) compared with those who stopped breastfeeding within 6 months postpartum.

**Limitations:**

Finer details such as pregnancy complications and delivery information may not be accurate since all results are based on questionnaire responses. There may be a sampling bias because women who suffer from postpartum hair loss may tend to participate more frequently.

**Conclusion:**

Over 90% of women experienced postpartum hair loss. Our data show that long-term breastfeeding and preterm labor correlate with postpartum hair loss.

**What is known about this subject with respect to women and their families?**

- Postpartum hair loss is believed to be common, and it is a diffuse alopecia that begins about 2–4 months after delivery and lasts for about 6 months to 1 year.
- Some information about postpartum hair loss can be found on the internet, but there is no research-based information about its frequency and timing.

**What is new in this article with respect to women and their families?**

- Obstetricians and women, in general, should be made aware of the fact that postpartum hair loss occurs in so many postpartum child-rearing women.
- Many women may cope better with postpartum hair loss if they were aware that over 90% of women who participated in our study had experienced postpartum hair loss.
- Women may feel some relief upon knowing the optimal duration of breastfeeding that can reduce postpartum hair loss.

## Introduction

Postpartum hair loss is a diffuse alopecia that begins about 2–4 months after delivery and lasts for about 6 months to 1 year.^1^ Although there are individual differences in its severity, some women have more hair loss across the entire scalp and some have hair loss for longer periods of time. This results in women refraining from social interactions or using a hat or a wig to be involved socially. Although women who feel anxious regarding postpartum hair loss require more awareness of the situation and appropriate information, there is very little reliable information available on this topic. For instance, even the prevalence of postpartum hair loss is not known.

Postpartum hair loss is thought to be a result of changes in the hair cycle that occur during pregnancy. The human hair follicle shows patterns of cyclic activity with periods of massive growth (anagen), apoptosis-driven involution (catagen), and a resting phase (telogen), and these phases last approximately 1,000, 10, and 100 days, respectively.^2,3^ The anagen phase is prolonged during pregnancy, and some follicles may even remain in the anagen phase during the entire pregnancy. All the overly active hair follicles enter the catagen phase simultaneously postpartum.^2–4^ This transition to the catagen phase is believed to result in excessive postpartum hair loss, and this type of alopecia is generally referred to as telogen effluvium.^3–5^ Telogen effluvium although a type of diffuse alopecia, is distinct from androgenetic alopecia, female-pattern hair loss, and alopecia areata.

In postpartum hair loss research conducted between the 1960s and 2010s, it has been speculated that postpartum hair loss may be caused by lower blood estrogen and progesterone levels, prolactin fluctuations, or thyroid hormone levels in the lactating mother.^2,6–8^ However, no studies have evaluated and assessed the relationship between blood hormone levels and the severity of postpartum hair loss.

In this study, we investigated several aspects of postpartum hair loss, including its frequency, timing, and impact on anxiety levels in postpartum child-rearing women participating in our study. Furthermore, we identified several factors that correlate with the pathogenesis of postpartum hair loss.

## Methods

### Design

The survey was conducted (The study protocol was reviewed and approved by the Tokyo Medical and Dental University Review Board and the Institutional Review Board of Tokyo Metropolitan Ohtsuka Hospital.) between June 2021 and April 2022. We included those postpartum child-rearing women who delivered at the 2 above-mentioned facilities and responded to our questionnaire 10–18 months after delivery. Those who had a history of alopecia before pregnancy and those with multiple pregnancies were excluded from this study.

The survey was conducted at the Department of Perinatal and Women’s Medicine of Tokyo Medical and Dental University Hospital and the Department of Obstetrics and Gynecology of Tokyo Metropolitan Ohtsuka Hospital between June 2021 and April 2022. At the beginning of web enrollment, a check box was provided to confirm their participation in the research study, and online electronic consent was obtained. The study was conducted in accordance with the Declaration of Helsinki.^9^ This study was registered with the University Hospital Medical Information Network (UMIN) in Japan, and the trial registration number is UMIN000042510 (Registration date: November 21, 2020).

### Protocol

The research description was sent by mail to all participants. A QR code and a URL were included in the research description, and the participants answered the questionnaire on the Internet. The questionnaire was anonymized using an ID. We sent the research description in 2 parts to adjust for the seasonal changes in hair loss. The interval between mailing the 2 parts was 8 months to avoid duplication of participants.

### Measures

In the questionnaire, the following questions were asked to assess the baseline characteristics: age at delivery, pregnancy and delivery history, height, prepregnancy weight, existing diseases, fertility treatments, current menstrual status, days from delivery to the resumption of menstruation, and smoking and alcohol intake before pregnancy and after delivery. Regarding pregnancy, delivery, and child care, we asked the following questions: delivery week, delivery method (vaginal delivery/elective cesarean section/emergency cesarean section), blood transfusion during labor, infant weight, abnormalities during labor, presence or absence of preterm labor, gestational diabetes mellitus, and hypertensive disorders of pregnancy, infant feeding at 3 months after delivery (breastfeeding/mixed feeding/formula feeding), and the duration of breastfeeding.

For postpartum hair loss, we first inquired about the extent of hair loss using a 4-point Likert scale (not at all, a little, quite a lot, very much). Furthermore, except for those who had no hair loss, the following questions were asked: start, peak, and end time of hair loss; whether they felt anxious or stressed about their hair loss (not at all, a little, quite a lot, very much); when they noticed their hair loss; whether they took any remedial action for their hair loss; and whether they talked to someone about their worries.

Sleep disorder was evaluated using the Athens insomnia scale (AIS), which was developed as a brief and easy-to-administer questionnaire for determining the severity of insomnia defined according to the International Classification of Disease Tenth Revision.^10,11^ Depressive symptoms were assessed using the Edinburgh postnatal depression scale (EPDS). The EPDS, originally developed in the United Kingdom in 1987, is a 10-item self-report measure designed to screen women for symptoms of emotional distress during the postpartum period using a 4-point Likert scale.^12^ EPDS was translated into Japanese in 1996, and its reliability and validity have been confirmed.^13^

### Statistical analyses

Detailed information on postpartum hair loss underwent initial analysis. We grouped those who had little, quite a lot, or very much postpartum hair loss together as a group with the presence of postpartum hair loss. We then compared the background characteristics and various parameters pertaining to pregnancy, delivery, and childcare between the 2 groups (presence and absence of hair loss) using univariate analyses (unpaired *t*-test, Mann–Whitney test, and Fisher’s exact test). We were able to identify the factors correlated with postpartum hair loss. Variables that emerged with possible prognostic value and variables that may impact postpartum hair loss were then entered into a multiple logistical regression analysis and a penalized likelihood approach called Firth logistic regression was used to identify parameters that were independently correlated with postpartum hair loss. Statistical analyses were performed using R version 4.2.0 (R Foundation for Statistical Computing, Vienna, Austria). A *P* value < 0.05 was considered significant.

## Results

**Fig. 1.**
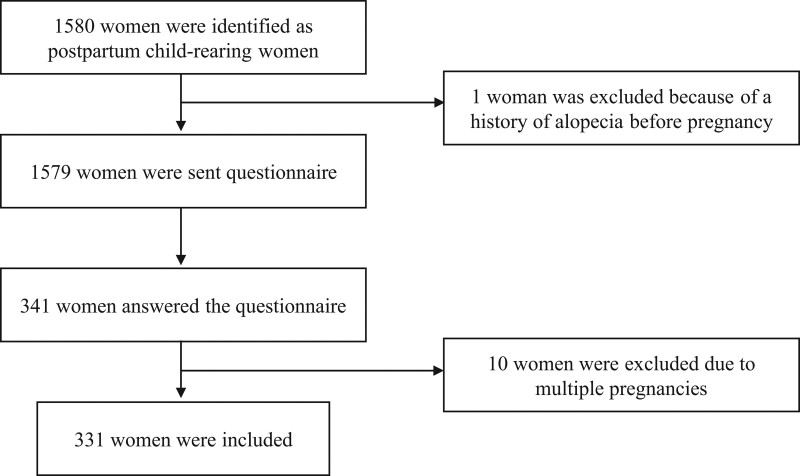
Flow diagram of study participants.

A total of 1,579 postpartum women received our questionnaire-based survey. Three hundred and forty-one (21.6%) women answered the questionnaire. Ten were excluded due to multiple pregnancies. Consequently, we analyzed the questionnaire responses from 331 participants (Fig. 1).

Detailed information on postpartum hair loss is shown in Table 1. Three hundred and four women (91.8%) reported the presence of at least some amount of postpartum hair loss, and 73.1% of these felt anxiety or stress about this condition. The average times for the start, peak, and end of hair loss were 2.9 months, 5.1 months, and 8.1 months, respectively. The majority of participants noticed hair loss at the time of washing their hair. In addition, 19.7% of women took remedial action to address their hair loss, and 61.7% of women talked to someone about their worries.

**Table 1:** Detailed information on postpartum hair loss

	Number (%)	Mean ± SD
Amount of postpartum hair loss (n = 331)^a^		
Not at all	27 (8.2)	
A little	102 (30.8)	
Quite a lot	154 (46.5)	
Very much	48 (14.5)	
Start time of hair loss, months after delivery^a^		2.9 ± 1.4
Peak time of hair loss, months after delivery^a^		5.1 ± 2.1
End time of hair loss, months after delivery^a^		8.1 ± 2.6
Did you feel anxious or stressed about hair loss? (n = 301)^b^		
Not at all	81 (26.9)	
A little	142 (47.2)	
Quite a lot	57 (18.9)	
Very much	21 (7.0)	
When did you feel hair loss? (n = 301)^c^		
While washing hair	271 (90.0)	
While setting hair	141 (46.8)	
When finding hair loss on the floor or pillow	139 (46.2)	
When someone pointed out	28 (9.3)	
Did you take any action against hair loss? (n = 300)^b^		
Yes	59 (19.7)	
No	241 (80.3)	
Did you talk to someone about your worries? (n = 298)^b^		
Yes	184 (61.7)	
No	114 (38.3)	

a Excluding those who had no hair loss.

b Excluding those who had no hair loss and who did not answer the question.

c Excluding those who had no hair loss and who did not answer the question; multiple answers allowed.

The baseline characteristics of the participating women are shown in Table 2. We divided the participants into 2 groups according to the absence or presence of postpartum hair loss and assessed their baseline characteristics and various parameters related to pregnancy, delivery, and childcare using univariate analyses (Table 2). The postpartum hair loss group had earlier delivery (39.0 vs. 38.0 weeks, *P* = .033), a lower birth weight (3,113 g vs. 2,909 g, *P* = .047), and a higher preterm labor rate (0% vs. 17.9%, *P* = .014). Among women who had ended breastfeeding (n = 139), women with hair loss had tended to breastfeed for a longer term (8.7 months vs. 10.6 months, *P* = .075). Variables identified by univariate analyses that may be correlated with hair loss and variables considered to affect postpartum hair loss in the literature were then entered into a multiple logistical regression analysis (Table 3). Specifically, we analyzed 4 factors: age at delivery, week of delivery, infant weight, and duration of breastfeeding. The duration of breastfeeding, given its impact on estrogen levels, was considered a clinically important factor with the potential of affecting postpartum hair loss. Hence, we included it in the multivariate analyses, even though there was no significant difference found in the univariate analysis. Regarding preterm labor, there were no women who underwent preterm labor among those who had no hair loss. We analyzed preterm labor using the Firth logistic regression that is appropriate for such instances. A multiple logistic regression analysis was performed on 271 women without preterm labor. Based on our analyses, the duration of breastfeeding was deemed to be an independent predictor of postpartum hair loss (Table 3, Model 1). Among women who terminated breastfeeding between 6 and 12 months, the adjusted odds ratio for experiencing postpartum hair loss was 5.96 (95% confidence interval [CI], [1.68, 21.09]) compared with those who stopped breastfeeding ≤6 months postpartum. On the other hand, the odds ratio for those women who breastfed ≥12 months was 6.37 (95% CI [1.95, 20.76]). These results did not change even after adjusting for other variables that could affect postpartum hair loss (Table 3, Model 2).

**Table 2:** Baseline characteristics of the participants (n = 331), and comparison between the absence and presence of postpartum hair loss group using univariate analyses

	No hair loss (n = 27)		A little or more hair loss (n = 304)		*P* value
	Mean ± SD	%	Mean ± SD	%	
Age at delivery, y	33.8 ± 3.9		34.6 ± 4.5		0.411^a^
Gravida, n	1.9 ± 1.2		2.3 ± 1.4		0.221^a^
Para, n	1.6 ± 0.8		1.7 ± 1.0		0.466^a^
Height, cm	158.0 ± 6.0		159.0 ± 5.4		0.377^a^
Prepregnancy weight, kg	55.2 ± 11.0		53.3 ± 8.7		0.292^a^
Pre-pregnancy BMI, kg/m^2^	22.0 ± 3.7		21.1 ± 3.1		0.140^a^
Delivery week	39.0 ± 1.3		38.0 ± 2.4		0.033^a^
Past history (yes/no)		4/23		60/244	0.799^b^
Preterm labor (yes/no)		0/27		52/252	0.012^b^
GDM (yes/no)		3/24		35/269	1.000^b^
HDP (yes/no)		1/26		24/280	0.707^b^
Fertility treatments (yes/no)		10/17		87/217	0.381^b^
Smoking before pregnancy (yes/no)		1/26		33/271	0.336^b^
Smoking after delivery (yes/no)		1/26		6/298	0.452^b^
Alcohol intake before pregnancy (yes/no)		14/13		187/117	0.411^b^
Alcohol intake after delivery (yes/no)		8/19		103/201	0.832^b^
Current menstruation (yes/no)		23/4		254/50	1.000^b^
Days from delivery to resumption of menstruation (n = 228)	206.1 ± 120.9		234.7 ± 121.5		0.325^a^
Infant feeding at 3 months after delivery					
Breastfeeding/mixed feeding/formula feeding		9/17/1		150/127/27	0.114^b^
Duration of breastfeeding, months (n = 139)	8.7 ± 4.7		10.6 ± 3.7		0.069^a^
Delivery method					
Vaginal delivery/elective cesarean section/emergency cesarean section		19/4/4		199/63/42	0.829^b^
Blood transfusion during labor (yes/no)		1/26		6/298	0.452^b^
Infant weight, g	3,112.9 ± 347.1		2,909.2 ± 519.7		0.047^a^
Abnormalities during labor (yes/no)		6/21		86/218	0.655^b^
AIS	6.4 ± 3.8		6.9 ± 4.4		0.607^a^
EPDS	6.1 ± 5.2		6.6 ± 5.8		0.690^a^

AIS, Athens insomnia scale; BMI, body mass index; EPDS, Edinburgh postnatal depression scale; GDM, gestational diabetes mellitus; HDP, hypertensive disorders of pregnancy.

a Unpaired *t*-test

b Fisher’s exact test

**Table 3:** Factors associated with postpartum hair loss according to multiple logistic regression analysis (n = 279)

	Crude OR (95% CI)	*P* value	Model 1 adjusted OR (95% CI)	*P* value	Model 2 adjusted OR (95% CI)	*P* value
Age at delivery, y	1.04 [0.96, 1.14]	0.342	1.02 [0.93, 1.12]	0.672	1.01 [0.91, 1.12]	0.833
Delivery week	0.76 [0.57, 1.03]	0.075	0.88 [0.62, 1.24]	0.455	0.92 [0.63, 1.35]	0.663
Infant weight, kg	0.37 [0.13, 1.06]	0.065	0.42 [0.12, 1.48]	0.178	0.39 [0.11, 1.42]	0.152
Duration of breastfeeding <6 months 6–12 months >12 months	Reference 3.85 [1.06, 13.91] 5.82 [1.93, 17.49]	0.040 0.002	Reference 4.75 [1.25, 18.08] 6.83 [2.13, 21.86]	0.022 0.001	Reference 5.57 [1.39, 22.31] 8.02 [2.33, 27.54]	0.015 <0.001
Para, n	1.14 [0.72, 1.79]	0.57			1.05 [0.62, 1.79]	0.852
Pre-pregnancy BMI, kg/cm^2^	1.00 [0.98, 1.02]	0.895			1.00 [0.98, 1.03]	0.785
GDM and/or HDP (yes/no)	1.25 [0.41, 3.79]	0.693			1.63 [0.45, 5.93]	0.456
Delivery method Vaginal delivery Elective cesarean section Emergency cesarean section	Reference 1.61 [0.53, 4.95] 1.03 [0.33, 3.20]	0.403 0.964			Reference 1.10 [0.29, 4.12] 0.64 [0.18, 2.32]	0.886 0.499
AIS	1.03 [0.93, 1.13]	0.571			1.01 [0.90, 1.14]	0.838
EPDS	1.01 [0.94, 1.09]	0.756			1.01 [0.92, 1.11]	0.800

AIS, Athens insomnia scale; BMI, body mass index; CI, confidence interval; EPDS, Edinburgh postnatal depression scale; GDM, gestational diabetes mellitus; HDP, hypertensive disorders of pregnancy; OR, odds ratio.

Similarly, preterm labor was analyzed using the Firth logistic regression (Table 4). The adjusted odds ratio for those women who had preterm labor to also have postpartum hair loss was 9.59 (95% CI [1.24, 1,235.69]) compared with those who did not have preterm labor. Regarding other variables, the duration of breastfeeding was correlated with postpartum hair loss, as described above.

**Table 4:** The Firth logistic regression performed for multivariate analysis including preterm labor (n = 331)

	Coefficients	OR (95% CI)	*P* value
Age at delivery, y	0.02	1.02 [0.93, 1.12]	0.669
Delivery week	−0.11	0.90 [0.63, 1.24]	0.538
Infant weight, kg	−0.81	0.45 [0.13, 1.51]	0.197
Duration of breastfeeding <6 months 6–12 months >12 months	Reference 1.50 1.90	Reference 4.46 [1.24, 16.48] 6.66 [2.11, 20.18]	0.022 0.002
Preterm labor (yes/no)	2.27	9.65 [1.25, 1243.65]	0.025

CI, confidence interval; OR, odds ratio.

## Discussion

In this questionnaire-based cross-sectional study, we assessed the prevalence of postpartum hair loss and the incidence of anxiety and stress in the women sampled by our survey. We found that delayed termination of breastfeeding and preterm labor were the only 2 factors correlated with postpartum hair loss, suggesting that this condition may be impacted by estrogen levels. Our data also allow a possible relationship between postpartum hair loss and inflammation to be considered.

Postpartum hair loss is generally considered to start 2–4 months after delivery and to last 6 months to 1 year.^1^ Although it is believed to be common, the actual prevalence has not been quantified^1^ and little is known about the mechanisms involved.^8^ A review article reporting contradictory findings of very low and undefined postpartum hair loss prevalence has prematurely concluded that postpartum alopecia does not exist.^14^

In this study, we carried out a detailed investigation of postpartum hair loss in postpartum breastfeeding women. This is the first study that clarifies the prevalence and timing of hair loss with research-based evidence. Using our data from this study, we can help spread awareness and provide information to address the anxiety in women regarding this condition.

By using multiple logistic regression analysis, we identified the duration of breastfeeding as an independent predictor of postpartum hair loss. Breastfeeding delays the resumption of normal ovarian cycles by disrupting the pulsatile release of gonadotropin-releasing hormone from the hypothalamus and luteinizing hormone (LH) from the pituitary.^15^ Disruption of pulsatile LH signals results in reduced estradiol production. The suckling stimulus prevents a normal preovulatory LH surge and the ovarian follicles fail to rupture and instead become cystic or atretic, resulting in anovulation and amenorrhea. When the suckling stimulus has been sufficiently reduced, a normal preovulatory LH surge occurs, ovulation ensues, and estrogen and progesterone levels return to their prepregnancy levels. As a consequence of this hormonal shift, menstruation begins. Of note, many studies have speculated that estrogen is associated with hair growth,^16,17^ and some reports have suggested that postpartum hair loss is caused by a decrease in estrogen levels.^4,7^ However, further research is required to confirm and validate these results. Our data are an important step in that direction because they provide research-based evidence supporting a relationship between postpartum hair loss and estrogen levels. A previous study has concluded that hormone replacement therapy was not effective for postpartum hair loss.^18^ However, based on our results, it may be possible to reduce postpartum hair loss by an early termination of breastfeeding for those women who suffer from this condition. There are individual differences in how breastfeeding women perceive postpartum hair loss. We also performed analyses by adjusting for EPDS and AIS, questionnaires based on subjective assessment, and yet there was no change in our results. Even by taking the differences in individual perception into account, we still believe that the obtained results are related to postpartum hair loss.

Additionally, our study also found that preterm labor was correlated with postpartum hair loss. In multiple logistic regression analysis, there was no relationship between the delivery week or infant weight and postpartum hair loss. Therefore, we believe that the pathology of preterm labor is correlated solely with postpartum hair loss. The presence or absence of premature labor in this study was determined by the responses to a questionnaire from the participants. This is a limitation because there is no specific information regarding whether the participants required treatment, rest at home, or hospitalization. However, inflammation is considered to be an important factor in preterm labor,^19^ and inflammation is also one of the causes of general hair loss.^20^ Inflammation during pregnancy might affect the hair growth cycle.

This study has two limitations. First, since all results are based on questionnaire responses, finer details such as pregnancy complications and delivery information may not be accurate, and blood tests including hormonal environment could not be analyzed. Second, there may be a sampling bias because women who suffer from postpartum hair loss may tend to participate more frequently in such questionnaires and also answer the questions compared with participants who received the questionnaire but had not experienced postpartum hair loss.

Further research is needed to investigate the relationship between postpartum hair loss and the duration of breastfeeding. As research on this problem advances, the relationship between postpartum hair loss and the duration of breastfeeding will become better understood and it will become possible to present the option of transitioning to formula feeding to women suffering from postpartum hair loss.

## Conclusion

Over 90% of women included in our study experienced postpartum hair loss. Long-term breastfeeding and preterm labor are associated with postpartum hair loss, suggesting that postpartum hair loss could be regulated by estrogen levels. A possible correlation of postpartum hair loss with inflammation was also considered in this study. Our data are an important step towards clarifying the cause of postpartum hair loss in the future and will serve as an opportunity to explore treatments for improving postpartum hair loss.

## Conflicts of interest

None.

## Funding

None.

## Study approval

The study protocol was reviewed and approved by the Tokyo Medical and Dental University Review Board and the Institutional Review Board of Tokyo Metropolitan Ohtsuka Hospital.

## Author contributions

AH: Participated in research design, performance of the research, the writing of the article, and data analysis. MT and NM: Participated in the research design, performance of the research, and data analysis. TO, AF, KT, MS: Participated in the performance of the research. MI: Participated in the research design and performance of the research. TA and KT: Participated in data analysis. All authors have seen and approved the final version.

## Data availability

Data that support the findings of this study are available from the corresponding author (AH) upon reasonable request.
